# Leishmanicidal and immunomodulatory properties of Brazilian green propolis extract (EPP-AF^®^) and a gel formulation in a pre-clinical model

**DOI:** 10.3389/fphar.2023.1013376

**Published:** 2023-02-09

**Authors:** Jéssica Rebouças-Silva, Nathaly Alcazar Amorim, Flávio Henrique Jesus-Santos, Jéssica Aparecida de Lima, Jonilson Berlink Lima, Andresa A. Berretta, Valéria M. Borges

**Affiliations:** ^1^ Laboratory of Inflammation and Biomarkers, Gonçalo Moniz Institute, Oswaldo Cruz Foundation, Salvador, Bahia, Brazil; ^2^ Faculty of Medicine of Bahia, Federal University of Bahia (UFBA), Salvador, Bahia, Brazil; ^3^ Laboratory of Research, Development and Innovation, Apis Flora Industrial e Comercial Ltda, Ribeirão Preto, São Paulo, Brazil; ^4^ Federal University of Western of Bahia (UFOB), Barreiras, Bahia, Brazil

**Keywords:** Leishmaniasis, propolis, neglected disease, natural products, *L. amazonensis*

## Abstract

Leishmaniasis is a widespread group of neglected vector-borne tropical diseases that possess serious therapeutic limitations. Propolis has been extensively used in traditional medical applications due to its range of biological effects, including activity against infectious agents. Here we evaluated the leishmanicidal and immunomodulatory properties of Brazilian green propolis extract (EPP-AF^®^) and a gel formulation incorporating EPP-AF^®^, in both *in vitro* and *in vivo* models of *Leishmania amazonensis* infection. Propolis extract, obtained from a standardized blend following hydroalcoholic extraction, showed the characteristic fingerprint of Brazilian green propolis as confirmed by HPLC/DAD. A carbopol 940 gel formulation was obtained containing propolis glycolic extract at 3.6% w/w. The release profile, assessed using the Franz diffusion cell protocol, demonstrated a gradual and prolonged release of *p*-coumaric acid and artepillin C from the carbomer gel matrix. Quantification of *p*-coumaric acid and artepillin C in the gel formulation over time revealed that *p*-coumaric acid followed the Higuchi model, dependent on the disintegration of the pharmaceutical preparation, while artepillin C followed a zero-order profile with sustained release. *In vitro* analysis revealed the ability of EPP-AF^®^ to reduce the infection index of infected macrophages (*p* < 0.05), while also modulating the production of inflammatory biomarkers. Decreases in nitric oxide and prostaglandin E_2_ levels were observed (*p* < 0.01), suggesting low iNOS and COX-2 activity. Furthermore, EPP-AF^®^ treatment was found to induce heme oxygenase-1 antioxidant enzyme expression in both uninfected and *L. amazonensis*-infected cells, as well as inhibit IL-1β production in infected cells (*p* < 0.01). ERK-1/2 phosphorylation was positively correlated with TNF-α production (*p* < 0.05), yet no impact on parasite load was detected. *In vivo* analysis indicated the effectiveness of topical treatment with EPP-AF^®^ gel alone (*p* < 0.05 and *p* < 0.01), or in combination with pentavalent antimony (*p* < 0.05 and *p* < 0.001), in the reduction of lesion size in the ears of *L. amazonensis-*infected BALB/c mice after seven or 3 weeks of treatment, respectively. Taken together, the present results reinforce the leishmanicidal and immunomodulatory effects of Brazilian green propolis, and demonstrate promising potential for the EPP-AF^®^ propolis gel formulation as a candidate for adjuvant therapy in the treatment of Cutaneous Leishmaniasis.

## Introduction

Leishmaniasis, a widespread group of neglected vector-borne tropical diseases caused by *Leishmania* spp. (protozoa: Trypanosomatidae), is characterized by pronounced epidemiological diversity and heterogeneous clinical features. Ranking among the top ten Neglected Tropical Diseases (NTD) due to its global impact on public health, the disease primarily affects poor countries in Southeast Asia, East Africa and Latin America (WHO, 2021).

The lack of commercial interest in developing new drugs to treat leishmaniasis has contributed to current treatment strategies becoming outdated. Despite moderate efficacy rates provided by current treatments, first- and second-line options present serious limitations, such as: High cost, considerable toxicity and parenteral route of administration in most cases, making treatment access difficult. In addition, increasing reports of therapeutic failure and parasite resistance are notable disadvantages (Romero et al., 2001; Carnielli et al., 2019; Kakooei et al., 2020; Kosaka et al., 2020; Silva et al., 2021).

Propolis is a resinous and waxy substance produced by *Apis mellifera* bees from exsudates and plant tissues. In Brazil, the great diversity of plant species visited by bees leads to significant variation in active ingredients. In all, 14 distinct types of propolis have been described in Brazil, classified according to botanical origin, physiochemical properties and geolocation (Park et al., 2002; Daugsch et al., 2008; Ferreira et al., 2017). Among the known types, Brazilian green propolis is one of the most commercialized and studied. Its main components are phenolic compounds, such as *p*-coumaric acid, caffeic acid, kaempferol, baccharin, drupanin and artepillin C, the latter being considered a marker of green propolis (de Sousa et al., 2007; Santos et al., 2020).

Standardized Brazilian green propolis extract is distinct from other propolis extracts in that its chemical composition and biological properties are consistent across production runs, enabling batch-to-batch reproducibility. A range of biological effects have been documented in therapeutic applications, including anti-ulcer, anti-inflammatory, antimicrobial, antioxidant benefits. More recently, a clinical study demonstrated the benefits of adjuvant treatment with propolis in patients hospitalized due to COVID-19, with significant reductions in the length of patient hospital stays (Berretta et al., 2013; 2012; Machado et al., 2012; Barud et al., 2013; Hori et al., 2013; Cusinato et al., 2019; Marquele-Oliveira et al., 2019; Diniz et al., 2020; Silveira et al., 2021).

Studies have demonstrated the leishmanicidal effect of different extracts of propolis both *in vitro* and *in vivo* (Devequi-Nunes et al., 2018; Regueira-Neto et al., 2018; Cavalcante et al., 2021). Previously, our group described the leishmanicidal and immunomodulatory effects of three different extracts of green propolis in *Leishmania braziliensis* infection *in vitro,* as well as directly on the promastigote forms of this parasite (Rebouças-Silva et al., 2017). Herein we build on our previous work by evaluating Brazilian green propolis extract in the context of *in vitro* and *in vivo L. amazonensis* infection. Importantly, this species is the other main etiological agent of cutaneous leishmaniasis in Brazil, and is strongly associated with diffuse leishmaniasis, a rare and difficult-to-treat form of CL. The present study investigated the capability of Brazilian green propolis extract to modulate the main inflammatory markers related to immunological control of *L. amazonensis* infection *in vitro*, as well as to influence disease outcome in mice topically treated with a gel propolis formulation.

## Materials and methods

### Material and reagents

Schneider’s insect medium, lipopolysaccharide (LPS), IFN-γ, resazurin sodium salt, the ornithine decarboxylase (ODC) antibody and BHT (butylated hydroxytoluene) were all obtained from SIGMA-Aldrich (St Louis, MO, United States). Lactate Dehydrogenase (LDH) Cytotoxicity Detection Kit and Nutridoma™-SP supplement were obtained from Roche Diagnostics GmbH (Sandhofer Strasse, Mannheim, Germany). Inactive fetal bovine serum (FBS), RPMI 1640 medium and RPMI 1640 medium without phenol red and penicillin were purchased from GIBCO (Carlsbad, CA, United States). Streptomycin, L-glutamine and PBS 10x were obtained from Invitrogen (Carlsbad, CA, United States). The superoxide dismutase activity assay, NS398 COX-2 inhibitor and PGE_2_ Elisa kits were purchased from Cayman Chemical Company (Ann Arbor, Michigan, United States). The MILLIPLEX^®^ kit based on Luminex^®^ xMAP^®^ technology was obtained from Merck (Darmstadt, Germany). A protease and phosphatase inhibitor cocktail, T-PERTM Tissue Protein Extraction Reagent and SuperSignal™ West Atto Ultimate Sensitivity Substrate Kit were purchased from ThermoFisher Scientific (Waltham, MA United States). Primary β-actin, pERK and ERK antibodies were obtained from Cell Signaling (Danvers, MA, United States), while Cox-2 and iNOS antibodies were purchased from Calbiochem (San Diego, CA, United States). The HO-1 antibody was purchased from Enzo Life Science (Farmingdale, NY, United States). Arginase antibody was obtained from R&D Systems (Minneapolis, MN, United States). Nrf2 antibody was obtained from Abcam (Cambridge, United Kingdom). Propylene glycol was purchased from Rudnik Comércio de Produtos Químicos Ltda. (Cotia, SP, BR). Carbomer 940 (Carbopol^®^) and sodium metabisulphite were obtained from Henrifarma Produtos Químicos (São Paulo, SP, BR). Potassium sorbate was purchased from Cosmoquímica Indl. E Coml. Ltda. (São Paulo, SP, BR). Disodium EDTA was obtained from Zílquimica Produtos para Laboratórios Ltda. (Ribeirão Preto, SP, BR). Castor oil was purchased from Volp Indústria e Comércio Ltda. (Osasco, SP, BR). Triethanolamine was obtained from Química Moderna Indústria e Comércio Eireli (Barueri, SP, BR).

### Brazilian green propolis (EPP-AF^®^) extract and gel (3.6% w/w)

Brazilian green propolis (EPP-AF^®^) was obtained from selected Brazilian propolis raw material following chemical evaluation by the technical team of Apis Flora Indl. Coml. Ltda (Ribeirão Preto/SP, Brazil). The EPP-AF^®^ blend was prepared, frozen and crushed for extraction *via* ethanol and purified water (7:3). The extraction process involved maceration and percolation steps to obtain ethanolic propolis extract. The extract solvent was then evaporated under controlled temperature and low pressure conditions, and the resulting soft extract propolis was solubilized using propylene glycol. The gel vehicle was formulated by dispersing carbomer 940 (Carbopol^®^) in purified water preserved with potassium sorbate, sodium metabisulphite, disodium EDTA and BHT (butylated hydroxytoluene). Propolis glycolic extract was dissolved in hydrogenated ethoxylated castor oil. This mixture was then incorporated in the carbomer gel, with sufficient triethanolamine added to achieve gelation.

### Chemical characterization

Brazilian green propolis (EPP-AF^®^) extract and gel were evaluated using an HPLC/DAD system (Shimadzu apparatus equipped with a CBM-20 A controller, a LC-20AT quaternary pump, an SPD-M 20 A diode-array detector, and Shimadzu LC solution software, version 1.21 SP1) coupled to a Shimadzu Shim-Pack CLC-ODS column (4.6 mm × 250 mm, 5 µm particle diameter, 100 Å pore diameter). The mobile phase consisted of methanol (HPLC grade) and a water-formic acid solution (0.1% v/v), pH 2.7 (A). The method consisted of a linear gradient of 20%–95% methanol over a period of 77 min at a flow rate of 0.8 mL/min. Detection was set at 275 nm, in accordance with a previously published protocol (Berretta et al., 2012). Samples were diluted in 5 mL of methanol in 10 mL volumetric flasks, subjected to sonication for 10 min and filled to volume with Milli-Q water. All samples were filtered through a 0.45 µm filter before analysis. The chemical references used were caffeic acid (Sigma-Aldrich, L: SLBZ6416), *p*-coumaric acid (Sigma-Aldrich, L: 091M119V), 3,5 dicaffeoylquinic acid (Phytolab, L. 3215), 4,5–dicaffeoylquinic acid (Phytolab, L. 9943), galangin (Sigma-Aldrich: BCCG2648), artepillin C (Phytolab, L: 111674647), as well as aromadendrin-4′-O-methyl ether, drupanin and baccharin previously isolated by De Sousa et al. (2007).

### Release study

An *in vitro* release study was performed using Franz diffusion cells at 37°C, with a cellulose membrane (10 mm MWCO 12000-14000) and carbomer-based propolis gel in the donor compartment. The receptor compartment was filled with phosphate buffer at 10 mmol·L^-1^ (pH 7.4) with an additional 133 mmol·L^-1^ sodium chloride and hydrogenated ethoxylated castor oil (Marquele-Oliveira et al., 2019). This receptor solution was prepared targeting “sink conditions,” remaining under constant agitation with a magnetic bar in a controlled bath at 37°C throughout the entire protocol. Receptor solution samples (500 μl) were collected at the following intervals: 2, 4, 6, 8, 24, 28, 32, and 48 h, with the same volume of fresh receptor solution replaced following sample collection. Dilution was accounted for in the quantification calculations of propolis biomarkers (Marquele-Oliveira et al., 2019).

The obtained samples were analyzed by HPLC/DAD in quadruplicate. Chromatographic conditions were established in accordance with the protocol described by Berretta et al. (2012). The evaluated biomarkers of propolis were *p*-coumaric acid and artepillin C, both detected at 275 nm.

### Ethics statement

Female BALB/c mice aged 6–8 weeks were obtained from the animal care facility at the Gonçalo Moniz Institute (IGM-FIOCRUZ), located in the city of Salvador, Bahia-Brazil. All animal experimentation was conducted in accordance with the Guidelines for Animal Experimentation established by the Brazilian Council for the Control of Animal Experimentation (CONCEA). The present study received approval from the local institutional review board (CEUA protocol no: 005/2020, IGM/FIOCRUZ).

The animal study was reviewed and approved by Animal Experimentation established by the Brazilian Council for the Control of Animal Experimentation (CONCEA). Gonçalo Moniz Institute, Oswaldo Cruz Foundation, Salvador, Bahia, Brazil.

### Parasites

Wild-type *L. amazonensis* (MHOM/BR88/BA125) were cultured in Schneider’s insect medium supplemented with 10% inactivated Fetal Bovine Serum (FBS), 100 U/mL penicillin, 100 μg/mL streptomycin and 2 mM L-glutamine in 25 cm^2^ flasks at 24°C for 6 days.

### Macrophage toxicity assay

Bone-marrow derived macrophages (BMDM) were obtained from BALB/c mice femurs and cultivated at 37°C under 5% CO_2_ for 7 days in RPMI medium supplemented with 20% FBS, 100 U/mL penicillin, 100 μg/mL streptomycin, 2 mM L-glutamine and 30% L929 cell culture supernatant (a source of macrophage colony-stimulating factor). Next, differentiated BMDM were harvested using cold saline solution. BMDM (1 × 10^5^/well) were plated on 96-well plates and cultured at 37°C under 5% CO_2_ in RPMI medium without phenol red, supplemented with 1% Nutridoma™-SP (instead of FBS) for 24 h. BMDM were then treated with Brazilian green propolis (EPP-AF^®^) at variable concentrations (3.12–200 μg/mL, serial dilution factor 1:2) at 37°C for 48 h. Next, the cell supernatants were collected and LDH activity was quantified using the LDH cytotoxicity kit (Roche), while BMDM were incubated for an additional 4 h in culture medium containing 10% v/v sodium resazurin salt. Uninfected BMDM cultures supplemented with RPMI medium (Medium) or Triton X-100 (2% v/v) were used as cell viability and death controls, respectively. Absorbance was read at 570 nm and 600 nm for the resazurin sodium assay and at 492 nm for the LDH assay by spectrophotometer (SPECTRA Max 190).

### Macrophage infection

To evaluate parasite viability following EPP-AF^®^ treatment, BMDMs were first isolated as described above and plated at 3 × 10^5^/well on 24-well plates containing glass coverslips, or at 1 × 10^5^/cells per well on 96-well plates. The macrophages were then infected (10:1) with stationary-phase *L. amazonensis* promastigotes for 4 h. Next, cell cultures were washed twice with saline solution and either treated with 25 μg/mL or varying concentrations (3.12–25 μg/mL, serial dilution factor 1:2) of Brazilian green propolis extract (EPP-AF^®^) for 48 h. To evaluate intracellular amastigote viability following EPP-AF treatment, coverslips were stained with hematoxylin and eosin to calculate the infection index (percentage of infected cells × mean number of amastigotes per cell), as determined by random counts of 200 cells/field on each glass coverslip under optical light microscopy. To evaluate parasite viability, after treatment the medium was replaced with 0.2 mL of supplemented Schneider’s insect medium and cells were cultured at 24^°^C for an additional 6 days, after which the number of viable parasites was determined by direct counting.

### Inflammatory mediator quantification

BMDMs (5 × 10^5^/well on 96-well plates) were stimulated or not with IFN-γ (100 ng/mL) overnight and infected with stationary-phase *L. amazonensis* promastigotes for 4 h. Next, macrophages were washed twice to remove any non-internalized parasites. In some experiments, *L. amazonensis-*infected macrophages were pretreated with 1 µM of NS398, a COX-2 inhibitor, or 10 µM of the HO-1 enzyme inhibitor, SnPP (Sigma) for 1 h. RPMI cell medium was then replaced and IFN-γ stimulation was reapplied together with 25 μg/mL of Brazilian green propolis (EPP-AF^®^) either alone or together with inhibitors for 48 h. Next, the cell culture supernatants were collected, cells were washed in cold PBS 1x and then lysed with an appropriate lysis buffer. Cell extracts were stored at −20°C. The Griess reaction was used to measure nitrite levels (as a proxy for nitric oxide levels) in cell supernatants, while PGE_2_ was measured using a PGE_2_ Elisa kit. Cytokines IFN-y, IL-10, IL-12p70, IL-13, IL-17α, IL-1β, IL-4, IL-6, and TNF-α were quantified in cell supernatants using a MILLIPLEX^®^ kit. All procedures were performed in accordance with manufacturer instructions.

### Evaluation of COX-2 and HO-1 enzyme inhibition

Macrophages at a density of 1 × 10^5^ were infected with *L. amazonensis* and pretreated with 1 µM of NS398, a COX-2 inhibitor, or 10 µM of the HO-1 enzyme inhibitor, SnPP (Sigma) for 1 h. Subsequently, cell cultures were treated with 25 μg/mL of EPP-AF^®^ together with the inhibitors. After 48 h of treatment, the culture supernatants were aspirated and the culture medium was replaced with Schneider’s medium, followed by incubation for 6 days at 26°C. Viable promastigotes recovered in culture supernatants were counted by light optical microscopy. In some experiments, a density of 3 × 10^5^ macrophages were cultured under glass coverslips and treated as described above. Finally, coverslips were stained with H&E to determine the numbers of amastigotes per cell and infected cells.

### Western blot analysis

IFN-γ-primed or not BMDMs (5 × 10^5^/well) were infected with stationary-phase *L. amazonensis* promastigotes and treated with 25 μg/mL Brazilian green propolis (EPP-AF^®^) for 15, 30 or 45 min (for MAPK signaling pathway analysis) or 48 h (for HO-1, Arginase, ODC, iNOS, and Cox-2 analysis). Next, cells were washed twice with 1x PBS and then lysed in RIPA (Radio Immuno Precipitation Assay) buffer supplemented with a cocktail of protease and phosphatase inhibitors. Protein extracts were resolved on SDS-PAGE and transferred to a nitrocellulose membrane. Next, after blocking with 5% non-fat dry milk in TBS with 0.1% Tween-20 (TBS-T), gels were incubated overnight with antibodies against Cox-2, iNOS, HO-1, Arginase, ODC, pERK, ERK, and β-actin, followed by secondary antibody labeling. All membranes were then washed thrice with 0.1% TBS-T and blots were developed using a SuperSignal™ West Atto Ultimate Sensitivity Substrate Kit. Bands were detected on a Luminescent Image Analyzer using Image Quant Las 4010 software (GE healthcare).

### Infection and treatment *in vivo*

Female BALB/c mice were challenged with 10 μL of saline solution containing 10^6^ stationary-phase *L. amazonensis* promastigotes in the dermis of the left ear, using a 30 G needle (BD Ultra-fine II^®^). Treatment was begun 3 weeks after infection, with animals divided into three experimental groups (*n* = 10): 1) Untreated controls; 2) Vehicle control (i.e., gel base without the addition of propolis) and iii) topical propolis gel treatment (EPP-AF^®^, 3.6% w/w). Topical treatment was performed through daily applications of an amount of gel sufficient to cover the entire lesion area with the aid of dental cotton until the conclusion of experimentation. Lesion development was monitored weekly for up to 12°weeks using an analog caliper (Kroeplin).

For combination experiments, mice were infected as described above and then randomly assigned into four experimental groups (*n* = 15): 1) Vehicle control (Vehicle); 2) Topical propolis gel treatment (EPP-AF^®^); 3) Sb^5+^ (Glucantime^®^, 50 mg/kg/day, intraperitoneal, 5 days/week for 5 weeks) (Sb^5+^); 4) Topical propolis gel treatment plus Sb^5+^ (i.p.) (EPP-AF^®^ + Sb^5+^). At 10 weeks post-infection, mice were euthanized by cardiac puncture, after which serum was collected in a tube not containing anticoagulant. In addition, mouse ears and draining lymph nodes (dLN) near the site of infection were aseptically removed and homogenized in supplemented Schneider’s medium. The collected homogenates were then serially diluted and seeded onto 96-well plates. Parasite loads were determined using a limiting-dilution assay as described by Titus et al. The number of viable parasites was determined using the lowest dilution at which promastigotes were able to grow after 2 weeks of incubation at 24°C in a BOD incubator. Furthermore, half of the ears from each experimental group were quickly frozen in liquid nitrogen. Subsequently, these tissue samples were macerated using liquid nitrogen and then solubilized with protein extraction buffer (T-PERTM Tissue Protein Extraction Reagent) supplemented with protease inhibitors. Finally, the tissue extract was centrifuged and the supernatant was collected and stored at −80°C. Inflammatory mediator production was analyzed in all samples as described above.

### Statistical analysis

Data are presented as one representative experiment out of three independent experiments performed in quintuplicate (mean ± SD), or a pool of three independent experiments performed *in vivo* with no less than seven animals/group (mean ± SEM) (number of experiments performed indicated in each respective Figure legend). Kruskal–Wallis non-parametric testing was employed for multiple comparisons, while comparisons between two groups were performed using by Mann–Whitney non-parametric testing. Results were considered statistically significant when *p* < 0.05. All analyses were performed using GraphPad Prism v 8.00 or JMP Pro (version 13.0.0).

## Results

### Chemical profile of Brazilian green propolis (EPP-AF^®^) extract and gel

Brazilian green propolis glycolic extract and its carbomer gel formulation were chemically characterized to evaluated safety and efficacy in the experimental models. EPP-AF^®^ gel was also evaluated with respect to its release profile, since propolis biomarkers possess different characteristics and the timing of delivery of some actives may result in different behaviors, potentially modifying the obtained biological results. Due to its specific chemical and antioxidant characteristics, *p*-coumaric acid (164.159 molecular weight), a compound with 18.3 mg/mL of solubility in water and with 1.46 of octanol partition coefficient, was selected as a phenolic compound for evaluation. Additionally, artepellin C (300.396 molecular weight), a prenylated compound and a commonly used biomarker of Brazilian green propolis, possesses very low water solubility and an octanol partition coefficient of 5.4, and was therefore also selected.

**FIGURE 1:**
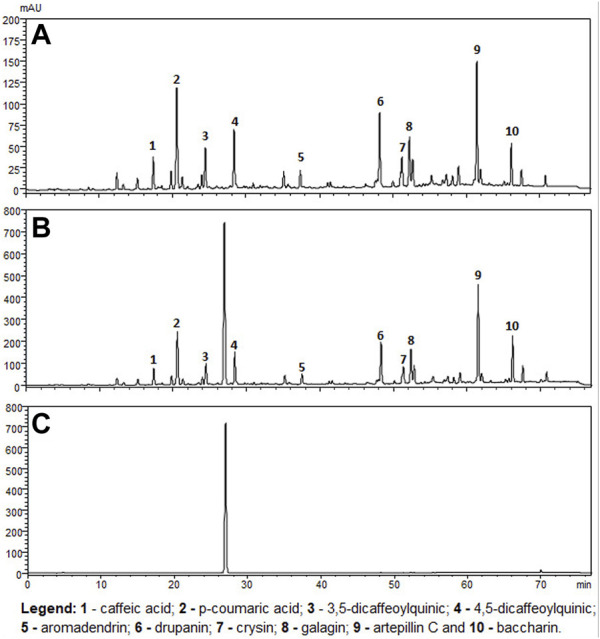
Chromatographic profiles (HPLC/DAD) of **(A)** EPP-AF^®^ glycolic propolis extract **(B)** carbopol gel containing propolis extract; **(C)** control sample of carbopol gel without propolis.

The chemical profile and quantitative results from evaluations of EPP-AF^®^ propolis extract (a), propolis gel (b) and carbomer gel vehicle c) are presented in Figure 1; Table 1, respectively. The gel vehicle containing only the carbomer and preserved purified water, without the addition of propolis extract, was evaluated to eliminate any possible signal interference that could affect the quantification of propolis biomarkers (Figure 1C).

**TABLE 1:** Chemical profile of EPP-AF^®^ propolis glycolic extract and carbomer gel using HPLC/DAD (*n* = 3, SD).

Propolis compounds	Chemical structure^a^	Propolis extract (mg/g)	Propolis gel (mg/g)
Caffeic acid	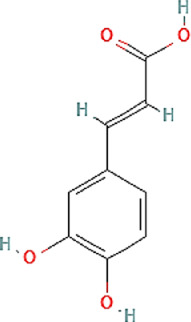	0.685 ± 0.028	0.098 ± 0.003
p-coumaric acid	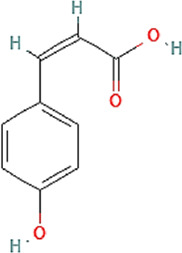	3.786 ± 0.027	0.496 ± 0.01
3,5-Dicaffeoylquinic acid	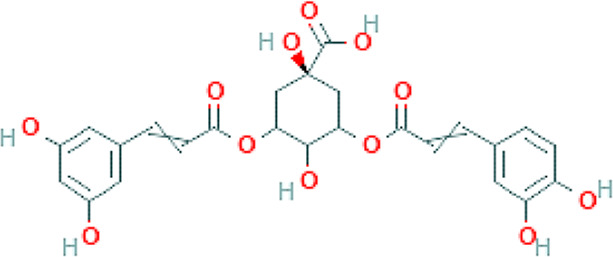	4.163 ± 0.006	0.574 ± 0.02
4,5-Dicaffeoylquinic acid	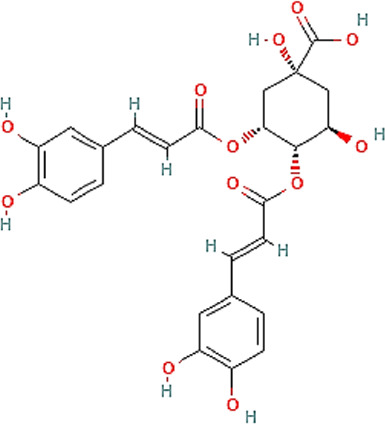	7.039 ± 0.074	0.959 ± 0.06
Aromadendrin-4′-O-methyl ether	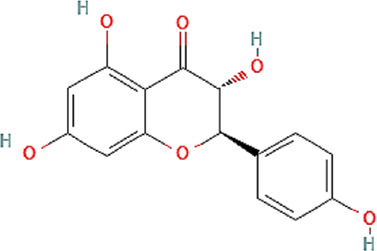	1.422 ± 0.016	0.208 ± 0.006
Drupanin	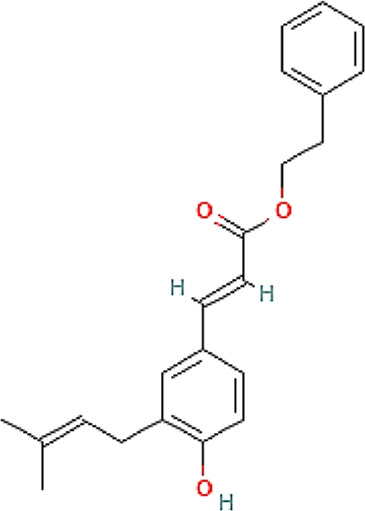	5.460 ± 0.123	0.750 ± 0.02
Chrysin	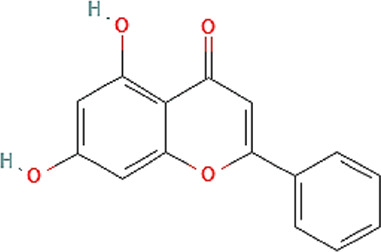	0.825 ± 0.025	0.125 ± 0.004
Galangin	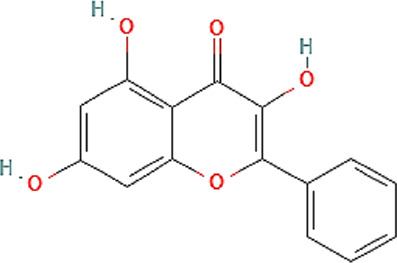	2.797 ± 0.188	0.479 ± 0.01
Artepellin C	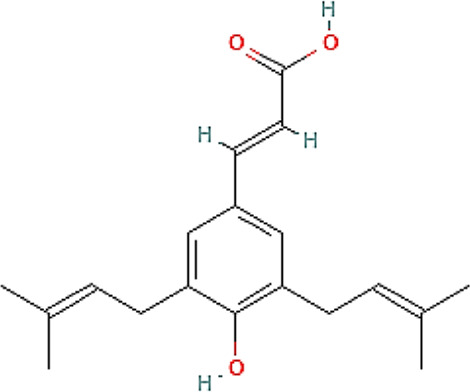	13.149 ± 1.647	2.630 ± 0.1
Baccharin	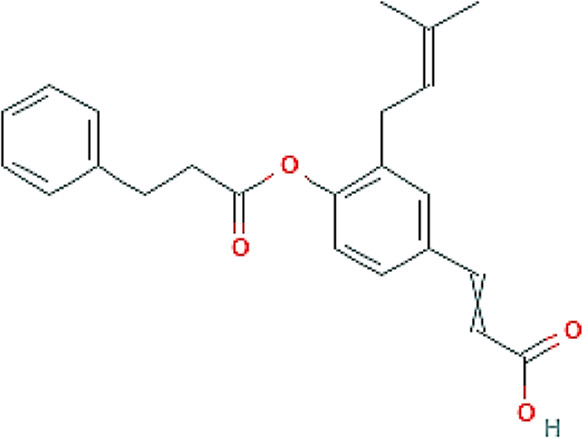	1.422 ± 0.264	0.378 ± 0.01

^a^ PUBCHEM, 2022.

### Brazilian green propolis EPP-AF^®^ gel presents a gradual and prolonged release profile

A release study was performed with EPP-AF^®^ gel using a Franz diffusion cell protocol to evaluate the controlled and sustained release of propolis biomarkers from the gel matrix, carried out for 48 h targeting “sink conditions.”

The previously obtained HPLC chromatographic profile was used to quantify the amount of actives released over time in accordance with the amount of EPP-AF^®^ gel applied in the donor compartment. The study was carried out with four replicates.

**FIGURE 2:**
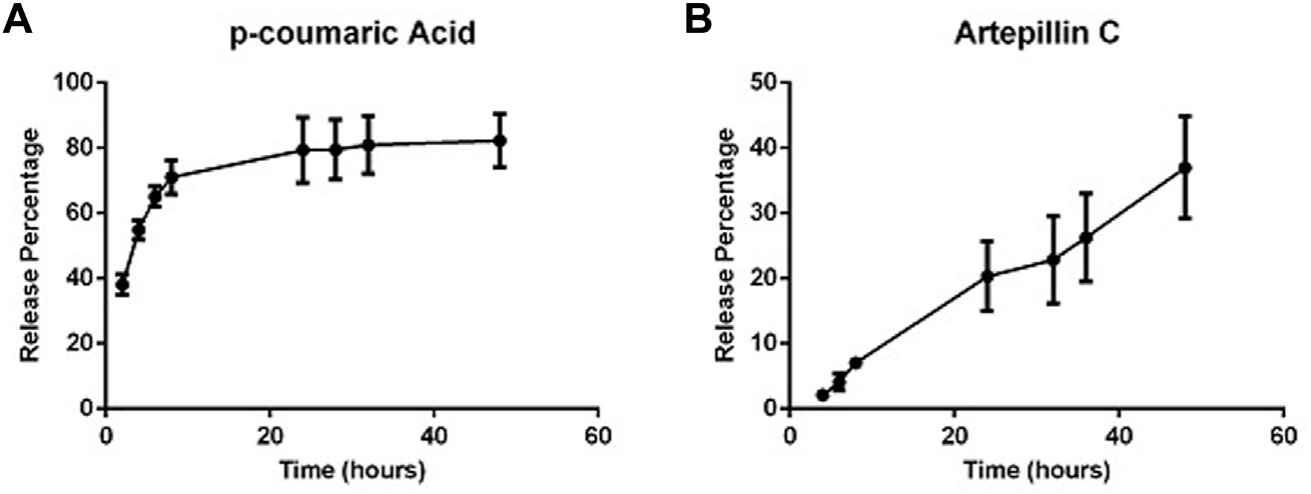
Release profile of propolis biomarkers from carbopol gel containing EPP-AF^®^ glycolic extract: **(A)** p-coumaric acid and **(B)** artepillin C.

Differences in the release profiles for each active can be explained by factors related to the carbomer gel release system and the physicochemical properties of the actives themselves. Our results showed that *p*-coumaric acid was released in greater amounts in the first hours compared to artepillin C (Figure 2), which can be explained by the hydrophilic characteristics of *p*-coumaric acid together with its lower molecular weight. By contrast, artepillin C possesses lipophilic properties, a higher partition coefficient and higher molecular weight, which delays its release from the gel matrix.

Overall, our release profile study demonstrated the absence of any “burst” effect, i.e., the gel release system used did not exhibit any immediate release of the evaluated actives, rather showing gradual and prolonged release from the carbomer gel matrix.

The kinetics of the release of the actives were also evaluated according to the zero-order, Higuchi model and first-order models considering the bulk amount of the active that was released during the study (Table 2). Correlation coefficient values indicate that the Higuchi model offered the best fit for the release of p-coumaric acid, as release was dependent on the square root of time.

**TABLE 2:** Release kinetics modeling with respective correlation coefficient r) values for *p*-coumaric acid and artepillin C actives.

Correlation coefficient values (r)			
Mathematical model		*p*-coumaric acid	Artepillin C
**Zero order**	(µg/cm^2^) h^-1^	0.812	0.995
**Higuchi Model**	(µg/cm^2^) ${\sqrt{h}}^{-1}$	0.882	0.990
**First Order**	Log (µg/cm^2^)h^-1^	0.754	0.936

The artepillin C biomarker showed different behavior in relation to its release kinetics. Despite very similar correlation coefficient values between the zero-order model and the Higuchi model, the kinetics of the zero-order model were predominant.

### Cytotoxicity of Brazilian green propolis (EPP-AF^®^) in uninfected BALB/c BMDM

**FIGURE 3:**
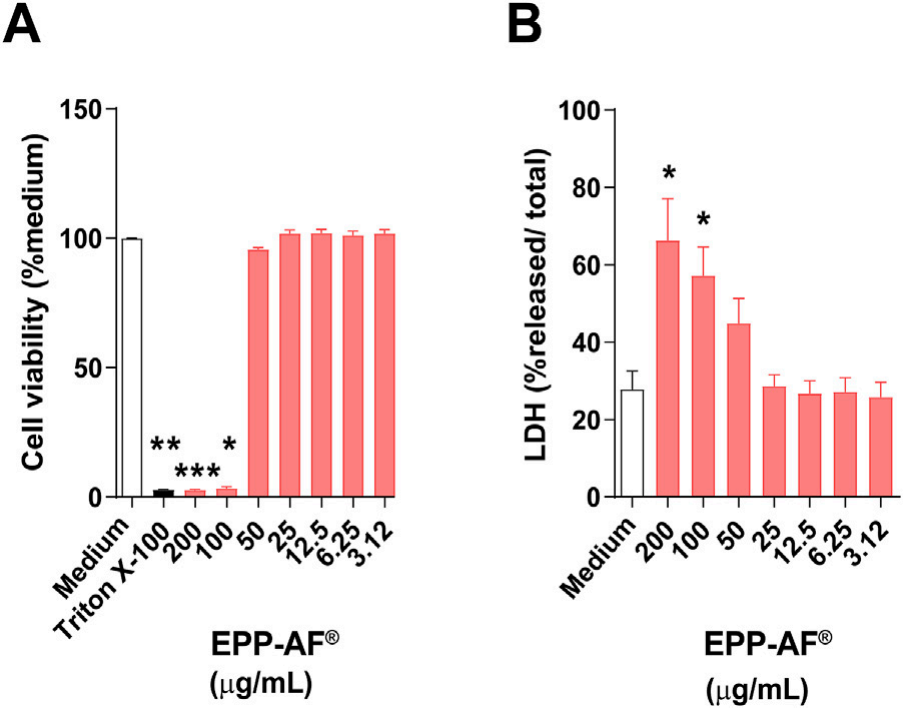
Cytotoxicity of EPP-AF^®^. The cytotoxic potential of EPP-AF^®^ was assessed by **(A)** Sodium resazurin reduction assay and **(B)** total LDH released after 48 h of treatment at varying concentrations (3.12–200 μg/mL). Bars represent ±SD of experiments performed in quintuplicate, representative of four independent experiments. The Kruskal-Wallis test, followed by Dunn’s post-test, were used for multiple comparisons (*p < 0.05, **p < 0.01, and ***p < 0.0001).

The cytotoxic effects of Brazilian green propolis (EPP-AF^®^) were assessed by resazurin reduction assay and by lactate dehydrogenase (LDH) activity in BMDM treated at concentrations of EPP-AF^®^ ranging from 3.12 to 200 μg/mL. After 48 h of treatment, concentrations greater than 100 μg/mL were found to significantly reduce cell viability by up 90% (*p* < 0.05 and *p* < 0.0001) (Figure 3A). Higher LDH release was also observed in cell cultures treated at >100 μg/mL (*p* < 0.05), indicating a loss of plasma membrane integrity (Figure 3B). Based on these results, concentrations at or below 25 μg/mL were established as suitable for the performance of all other assays.

### Brazilian green propolis (EPP-AF^®^) reduces *L. amazonensis* intracellular viability

**FIGURE 4:**
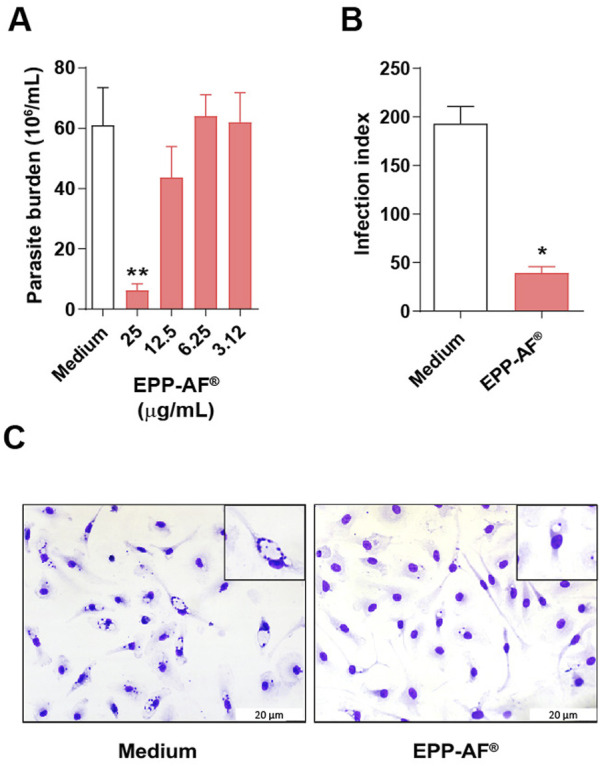
Leishmanicidal effect of EPP-AF^®^. BMDM were infected with *L. amazonensis* and treated at varying concentrations of EPP-AF^®^. **(A)** Concentration-response curve; **(B)** Infection index of *L. amazonensis-*infected macrophages after 48 h of treatment with 25 μg/mL green propolis extract; **(C)** Representative photographs of *L. amazonensis*-infected cells cultured with either medium alone or 25 μg/mL of green propolis extract and stained with H&E (40 × magnification). Bars represent ± SD of experiments performed in quintuplicate, representative of three independent experiments. The Kruskal-Wallis test, followed by Dunn’s post-test, were used for multiple comparisons (*p < 0.05 and **p < 0.01).

Next, the efficacy of Brazilian green propolis (EPP-AF^®^) to reduce intracellular *L. amazonensis* viability was evaluated. At 6 days after medium replacement, viable promastigote counts in cell culture supernatants were significantly lower (89%) compared to controls (*p* < 0.01) (Figure 4A). In cells treated with 25 μg/mL of EPP-AF^®^, a 79% reduction in the infection index was observed (*p* < 0.05) (Figure 4B). Representative images reveal intensely infected macrophages in cultures treated with RPMI medium alone, while fewer infected cells and amastigotes per cell were observed in cultures treated with 25 μg/mL of EPP-AF^®^ (Figure 4C). The IC_50_ value calculated for EPP-AF^®^ was 23 ± 4 μg/mL, with a corresponding Selective Index value of 3.07. However, treatment with Glucantime^®^ at concentrations ranging from 10 to 1,000 μg/mL did not evidence efficacy (data not shown).

For all subsequent macrophage experimentation and analysis, the EPP-AF^®^ treatment concentration was standardized at 25 μg/mL due to statistical significance regarding its effectiveness in reducing intracellular parasite viability.

### HO-1 expression induced by *in vitro* Brazilian green propolis (EPP-AF^®^) treatment

**FIGURE 5:**
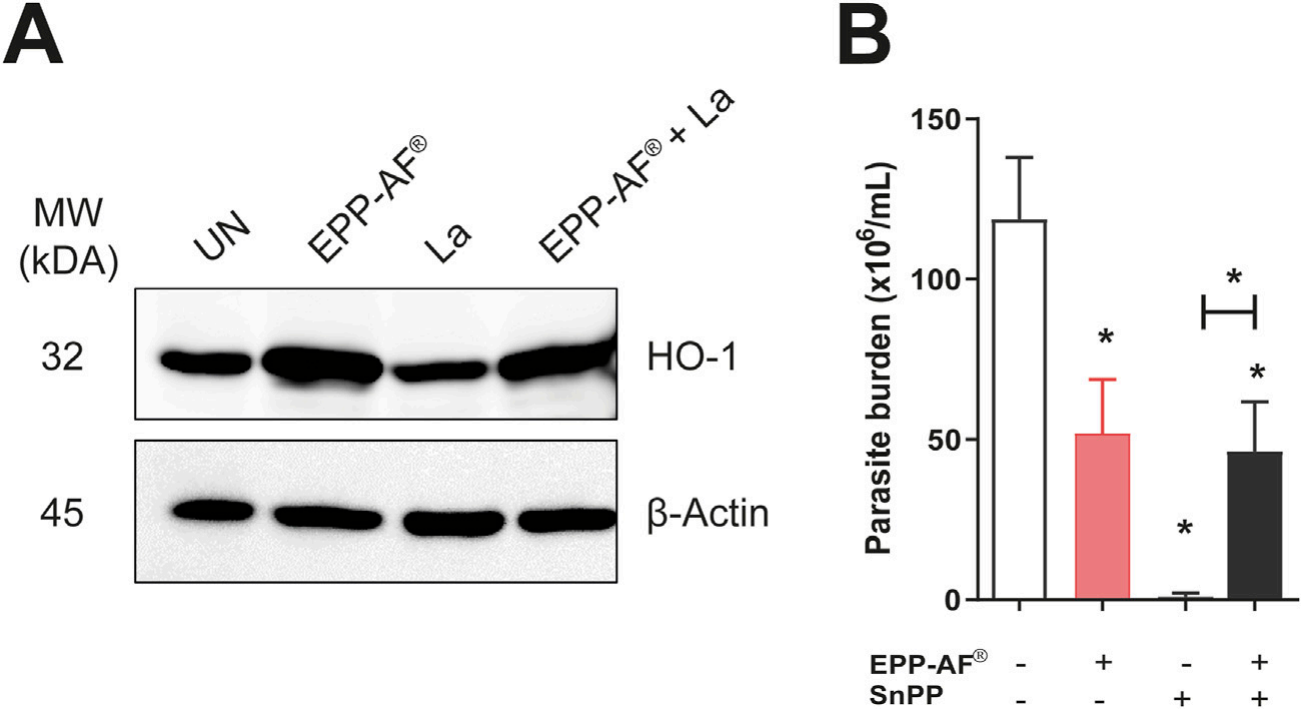
EPP-AF^®^ treatment induces HO-1 expression. BMDM were infected with *L. amazonensis* and treated with 25 μg/mL of EPP-AF^®^ as described in *Materials and Methods*. After 48 h, cell extract was collected to evaluate protein levels of **(A)** HO-1 by western blotting, with anti-β-Actin antibody used as an endogenous control. In a parallel assay, after 48 h of treatment, the culture medium was changed and **(B)** viable promastigotes were counted after 6 days in the presence or absence of the heme oxygenase inhibitor tin protoporphyrin (SnPP) and/or propolis. In **(A)**, Western blotting results are presented as a single experiment performed in quintuplicate, representative of three independent experiments. In **(B)**, bars represent mean parasite burden (± SD) from experiments performed in quintuplicate, representative of two independent experiments. The Kruskal-Wallis test, followed by Dunn’s post-test, were used for multiple comparisons, while the Mann-Whitney test was employed to compare two groups (*p < 0.05). La: *Leishmania amazonensis*; UN: Uninfected; EPP-AF^®^: Brazilian green propolis extract at 25 μg/mL.

Treatment with Brazilian green propolis (EPP-AF^®^) was found to induce the expression of HO-1 enzyme in both uninfected and infected *L. amazonensis* BMDM (Figure 5A). To validate the induction of HO-1 expression observed by Western blotting, infected macrophages were previously incubated with tin protoporphyrin IX (SnPP), an HO-1 inhibitor, and subsequently treated or not with 25 μg/mL of EPP-AF^®^. A significant reduction in the number of viable promastigotes recovered in the supernatants of cell cultures previously exposed to the SnPP inhibitor was observed (*p* < 0.05). Furthermore, EPP-AF^®^ treatment was observed to reverse the leishmanicidal effect of HO-1 inhibition (*p* < 0.05), yet without any statistical differences compared to cells treated with propolis extract alone (Figure 5B). These results suggest that while Brazilian green propolis (EPP-AF^®^) induces the expression of HO-1 by uninfected or infected *L. amazonensis* macrophages, it does not favor intracellular parasite survival.

### Brazilian green propolis (EPP-AF^®^) modulates iNOS and COX-2 enzyme expression, without impacting arginase 1

**FIGURE 6:**
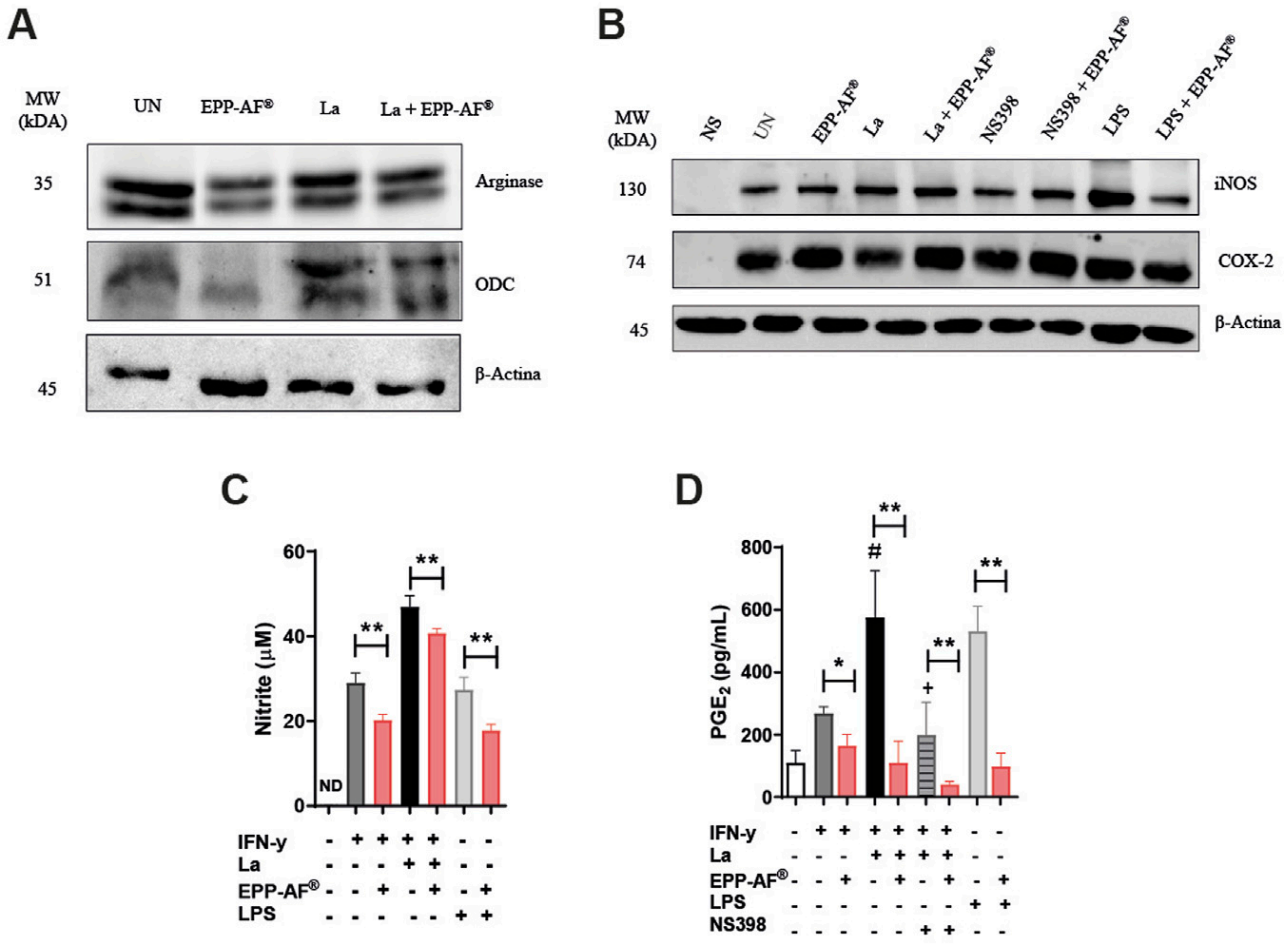
EPP-AF^®^ modulates iNOS and COX-2 enzyme expression, without impacting in arginase or ODC. BMDM were infected or not with *L. amazonensis,* stimulated or not with IFN-γ, and treated with EPP-AF^®^ at 25 μg/mL, as described in *Materials and Methods*. After 48 h of treatment, the cell extract was collected to evaluate protein expression by Western Blotting. **(A)** Arginase and ornithine decarboxylase (ODC), **(B)** iNOS and COX-2 (anti-β-Actin antibody used as an endogenous control). The levels of **(C)** nitrite and **(D)** PGE_2_ were evaluated in cell culture supernatants. Bars represent ±SD of experiments performed in quintuplicate, representative of three or one independent experiment(s). Western blotting results are presented as an experiment performed in quintuplicate, representative of two (Arginase-1 and ODC) or three (iNOS and COX-2) independent experiments. The Kruskal-Wallis test, followed by Dunn’s post-test, were used for multiple comparisons (*p < 0.05; **p < 0.01; #*p* < 0.05 in the comparison of uninfected condition (light gray bar) versus infected (black bar); +*p* < 0.05 in the comparison of the NS398 exposure condition versus levels of PGE2 in infected cells. La: *Leishmania amazonensis*; NS: Not Stimulated; UN: Uninfected and IFN-y stimulated; EPP-AF^®^: Brazilian green propolis extract at 25 μg/mL.

Treatment with Brazilian green propolis (EPP-AF^®^) was not found to modulate the expression of enzymes arginase 1 or ODC (Figure 6A), which must be considered a preliminary finding that warrants confirmation through additional experimentation. On the other hand, EPP-AF^®^ was shown to reduce the expression of iNOS exclusively in LPS-activated cells (Figure 6B). The quantification of nitrite levels (as indicator of nitric oxide levels) revealed that treatment with Brazilian green propolis EPP-AF^®^ significantly reduced the nitrite levels produced by BMDM in different inflammatory contexts, i.e., by LPS-activated cells as well as by uninfected and infected INF-γ-activated macrophages (*p* < 0.01) (Figure 6C).

While Brazilian green propolis EPP-AF^®^ seemed to subtly increase the levels of COX-2 expression by both uninfected and infected INF-γ-activated cells (Figure 6B), it unexpectedly reduced PGE_2_ levels expressed by LPS-activated cells, as well as by both uninfected and *L. amazonensis*-infected INF-γ-activated cells, in a significant manner (*p* < 0.01). Interestingly, propolis extract treatment seemed to potentiate the effect of the NS398 inhibitor in infected cells by further reducing PGE_2_ levels (*p* < 0.01) (Figure 6D).

### Brazilian green propolis (EPP-AF^®^) modulates cytokine production

**FIGURE 7:**
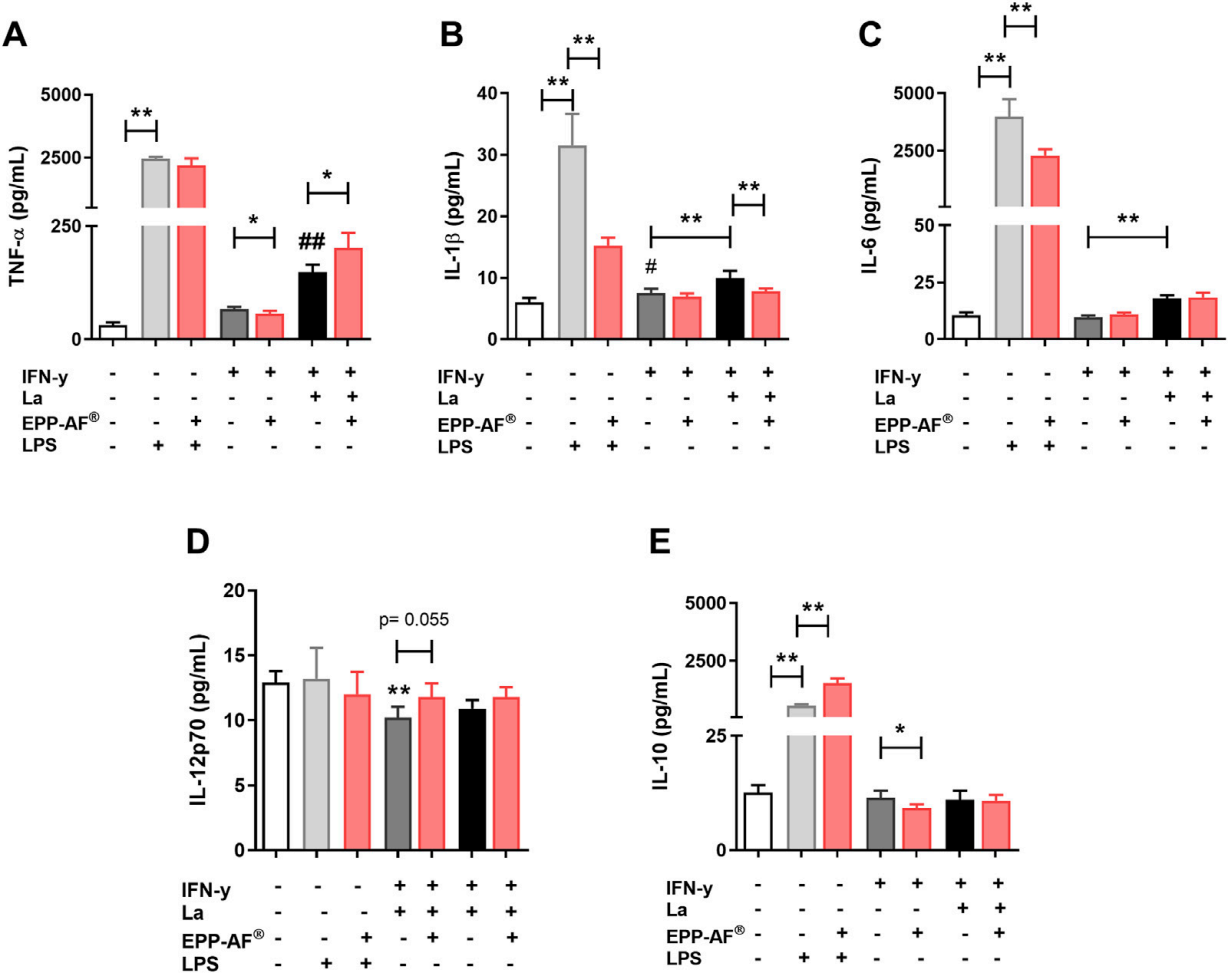
Immunomodulatory effect of EPP-AF^®^. BMDM, previously primed overnight with 100 U/mL IFN-y, were exposed to LPS (50 μg/mL) or infected with *L. amazonensis* and treated with EPP-AF propolis extract at 25 μg/mL. After 48 h of treatment, the supernatants were collected and the levels of cytokines **(A)** TNF-α; **(B)** IL-1β; **(C)** IL-6; **(D)** IL-12p70 and **(E)** IL-10 were evaluated by multiplex assay as described in *Materials and Methods*. Bars represent ± SD of experiments performed in quintuplicate. The Kruskal-Wallis test, followed by Dunn’s post-test were used for multiple comparisons, with Mann-Whitney testing applied for comparisons between two groups (*p < 0.05; **p < 0.01). For comparisons of TNF-α **(A)** and IL-1β **(B)** levels between uninfected (dark gray bar) and infected (black bar) conditions, ^#^ *p* < 0.05; ^##^ *p* < 0.01. La: *Leishmania amazonensis*.

The effect of propolis on cytokines produced by uninfected or *L. amazonensis-*infected IFN-y-activated macrophages was evaluated by multiplex assay, as described in *Materials and Methods* section. Brazilian green propolis (EPP-AF^®^) was shown to significantly increase the levels of TNF-α produced by infected cells (*p* < 0.05), yet reduced the levels of this mediator in uninfected IFN-y-primed cells (Figure 7A). On the other hand, treatment with propolis significantly reduced the production of the inflammatory cytokine IL-1β by *L. amazonensis-*infected cells (*p* < 0.01) (Figure 7B). No significant differences were observed regarding cytokines IL-6 (Figure 7C), IL-12p70 (Figure 7D) or IL-10 (Figure 7E), despite an increasing trend for IL-12p70 (*p* < 0.055). Furthermore, propolis extract attenuated the inflammatory profile of LPS-activated macrophages, as evidenced by decreased levels of inflammatory cytokines IL-1β (Figure 7B) and IL-6 (Figure 7C), as well as increased production of the regulatory cytokine IL-10 (Figure 7E).

### EPP-AF^®^ induces ERK 1/2 activation in uninfected and *L. amazonensis-*infected macrophages

**FIGURE 8:**
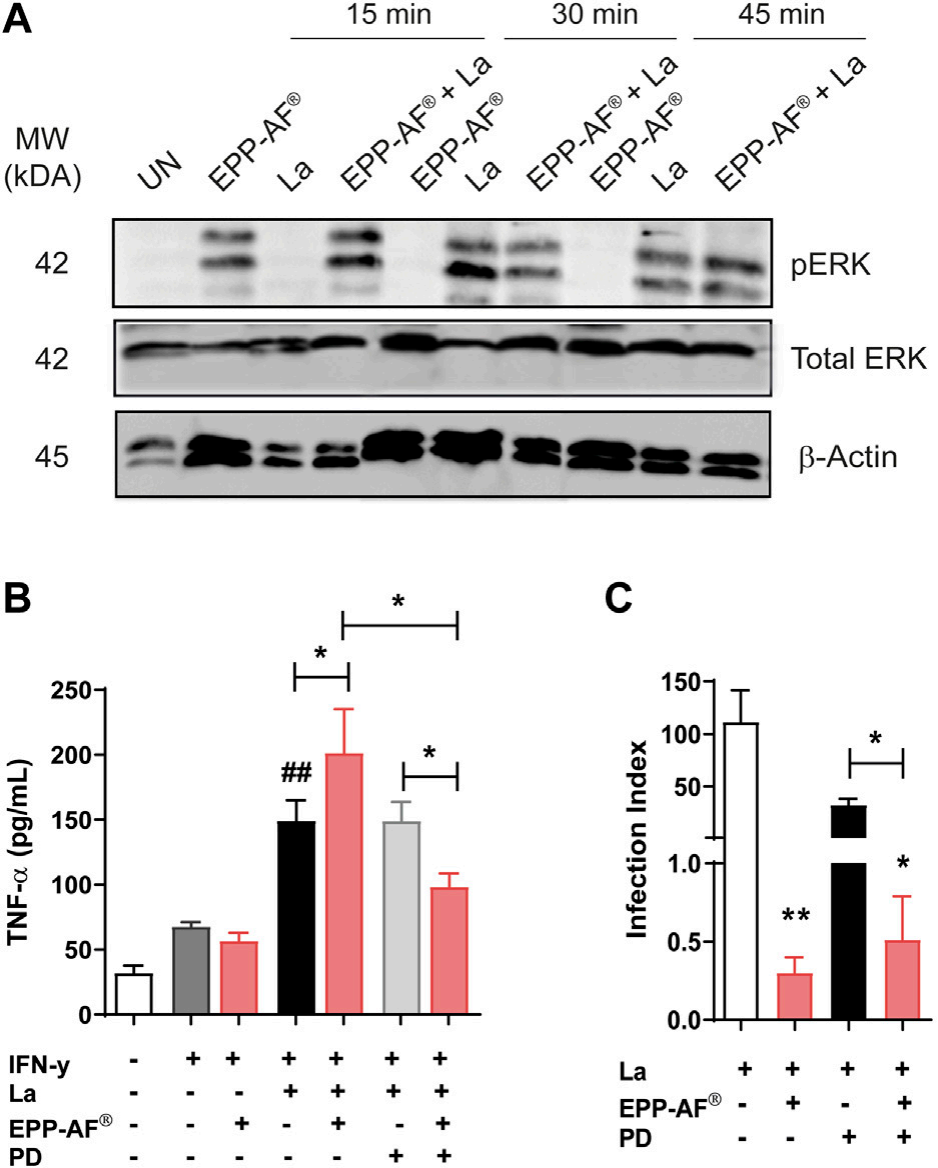
EPP-AF^®^ treatment activates ERK 1/2. BMDM were infected or not with *L. amazonensis* and treated with EPP-AF propolis extract at 25 μg/mL for 15, 30 or 45 min. After each treatment time, cell extracts were collected and analyzed by Western Blotting to evaluate the protein levels of **(A)** phosphorylated (pERK) and total (Total ERK) ERK. Anti-β-Actin antibody was used as an endogenous control. Levels of **(B)** TNF-α produced in the presence or absence of the ERK 1/2 inhibitor (PD98059) and/or propolis and **(C)** infection rate obtained under the same conditions. Bars represent ± SD of a representative experiment out of two independent experiments, performed in quintuplicate (Western blotting assay) or one representative experiment performed in quintuplicate (TNF-α and parasite burden assays). The Kruskal-Wallis test, followed by Dunn’s post-test were used for multiple comparisons, and Mann-Whitney testing was applied for comparisons between two groups (*p < 0.05 and **p < 0.01); For comparisons of TNF-α **(C)** levels between uninfected (dark gray bar) and infected (black bar) conditions, ^##^ *p* < 0.01. La: *Leishmania amazonensis*, UN: Uninfected, PD: PD98059 and EPP-AF^®^: Brazilian green propolis extract at 25 μg/mL.

Our findings suggest that Brazilian green propolis (EPP-AF^®^) activated the ERK 1/2 kinase in macrophages infected or not with *L. amazonensis* after 15 min of treatment. After 30 min of treatment, the expression of phosphorylated ERK 1/2 was detected in infected cells regardless of propolis treatment, remaining stable at 45 min post-treatment (Figure 8A). Subsequently, we evaluated whether the pharmacological inhibition of ERK 1/2 with the PD98059 inhibitor would influence the expression of inflammatory mediators following treatment with propolis. The PD98059 inhibitor produced relatively few changes in the observed cytokine profile of *L. amazonensis*-infected cells treated with propolis, notably a significant reduction in TNF-α levels (*p* < 0.05) (Figure 8B).

Since ERK 1/2 inhibition significantly reduced the production of TNF-α by *L. amazonensis*-infected cells treated with propolis, we then evaluated whether the inhibition of this pathway prior to treatment would influence the leishmanicidal effect associated with EPP-AF^®^. No significant differences were observed in the infection index of cells treated with propolis compared to treated macrophages previously exposed to the PD98059 inhibitor (Figure 8C).

Our data suggest that *in vitro* treatment with Brazilian green propolis (EPP-AF^®^) results in ERK 1/2 activation in association with enhanced TNF-α production by *L. amazonensis*-infected BMDM.

### Topical treatment with EPP-AF^®^ gel formulation reduces lesion size in *L. amazonensis*-infected mice

**FIGURE 9:**
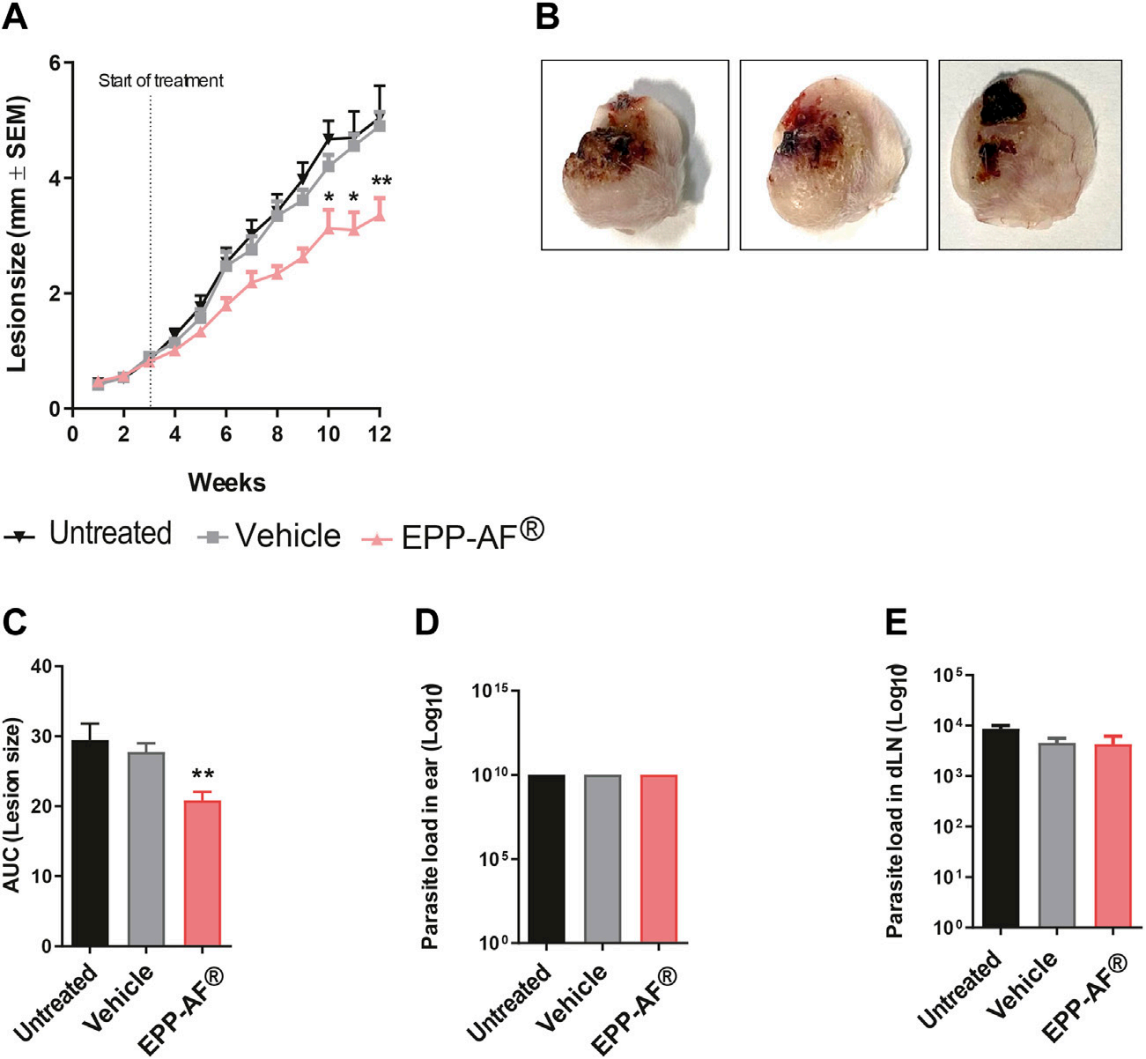
*In vivo* effect of topical treatment with EPP-AF^®^ gel on *L. amazonensis* infection. BALB/c mice were infected in the ear dermis with metacyclic *L. amazonensis* promastigotes and treated daily with EPP-AF^®^ propolis gel, as described in *Materials and Methods*. Lesion development **(A)** was evaluated weekly using an analog caliper. At the end of the 12th week of infection, the animals were euthanized, and the ears were photographed **(B)**. **(C)** Analysis of area under the ROC curve (AUC) corresponding to lesion size. Parasite load in the **(D)** ear and **(E)** draining lymph nodes was evaluated by limiting dilution technique. Bars represent ±SEM of a representative experiment involving 12 animals per group, from three independent experiments. The Kruskal-Wallis test, followed by Dunn’s post-test were used for multiple comparisons (*p < 0.05 and **p < 0.01). EPP-AF^®^: Brazilian green propolis gel.

Due to the promising results obtained from *in vitro* experimentation, the effectiveness of topical treatment with Brazilian green propolis gel was assessed in an experimental model of *L. amazonensis* infection. BALB/c mice were inoculated in the ear dermis with stationary phase *L. amazonensis*; after 3 weeks, EPP-AF^®^ (3.6% w/w) was applied topically daily. After 7 weeks of treatment, lesion size was significantly smaller at weeks 10 (*p* < 0.05), 11 (*p* < 0.05) and 12 (*p* < 0.01) in treated mice (Figure 9A), as additionally evidenced by calculating the area under the curve (AUC) (Figure 9C). Representative photographs of infected ears from each experimental group (Figure 9B) demonstrate reduced edema and a smaller ulceration area in the animals treated with EPP-AF^®^. Importantly, topical treatment was not observed to reduce parasite load in either the ear (site of infection and treatment) or the draining lymph nodes (Figures 9D, E, respectively). As expected, the application of the pharmaceutical vehicle control (i.e., gel base without the addition of propolis) had no effect on lesion size (Figure 9).

### Evaluation of combination therapy (EPP-AF^®^ gel and meglumine antimoniate) in *L. amazonensis* infection *in vivo*

**FIGURE 10:**
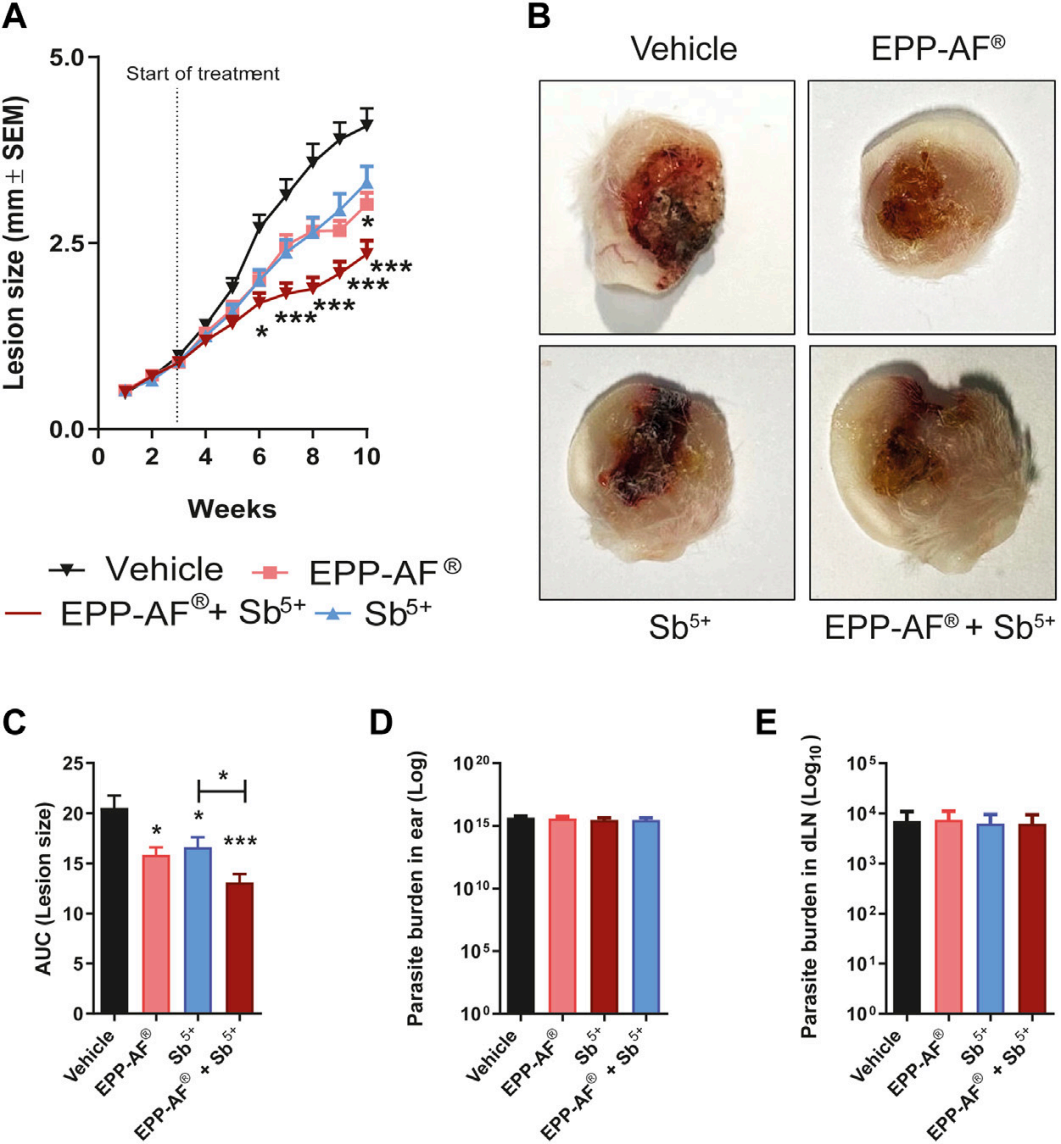
Synergistic effect of combined treatment (EPP-AF^®^ gel and meglumine antimoniate) in *L. amazonensis* infection *in vivo*. BALB/c mice were infected in the ear dermis with metacyclic *L. amazonensis* promastigotes and treated daily with EPP-AF^®^ gel. Lesion development **(A)** was evaluated weekly using an analog caliper. At the end of the 12th week of infection, the animals were euthanized and the ears were photographed **(B)**. **(C)** Analysis of area under the ROC curve (AUC) corresponding to lesion size. Parasite load in the **(D)** ear and **(E)** draining lymph nodes was evaluated by limiting dilution technique. Bars represent ±SEM of a pool of three experiments performed using at least 10 animals per group. The Kruskal-Wallis test, followed by Dunn’s post-test were used for multiple comparisons and the Mann-Whitney test was applied for comparisons between two groups (*p < 0.05 and ***p < 0.0001). EPP-AF^®^: Brazilian green propolis gel; Sb^5+^: meglumine antimoniate.

Considering the effectiveness of topical treatment with Brazilian green propolis gel, we endeavored to evaluate the combined effectiveness of EPP-AF^®^ in conjunction with meglumine antimoniate (Sb^5+^), the first-line treatment for leishmaniasis in Brazil. Weekly monitoring of lesion development demonstrated the effectiveness of combined treatment (EPP-AF^®^ + Sb^5+^), as significantly smaller lesions were observed from the sixth week after infection on (third week of treatment) compared to the pharmaceutical vehicle control (*p* < 0.05 and *p* < 0.001) (Figure 10A). Surprisingly, no significant differences were seen in response to treatment with Sb^5+^ alone when employing multiple comparisons testing (Kruskal-Wallis test, followed by Dunn’s post-test). However, statistical significance was observed using Sb^5+^ alone in comparison to the vehicle control (*p* = 0.024) under statistical testing for comparisons between two groups (Mann-Whitney). Non-etheless, the experimental group treated with EPP-AF^®^ gel alone again demonstrated a small, but statistically significant, reduction in lesion size at the 10th week post-infection (*p* < 0.05), corroborating previous experimental results (Figure 9A). Representative photographs of infected ears from each experimental group (Figure 10B) reveal that all modalities of treatment resulted in varying degrees of edema reduction and/or lesser extent of ulceration compared to the vehicle control group (Vehicle). Again, none of the treatments applied significantly impacted parasite load in either animal ears or draining lymph nodes (Figures 10D, E, respectively).

### Quantification of inflammatory mediators both systemically and at the site of infection following topical treatment with EPP-AF ^®^ gel

**FIGURE 11:**
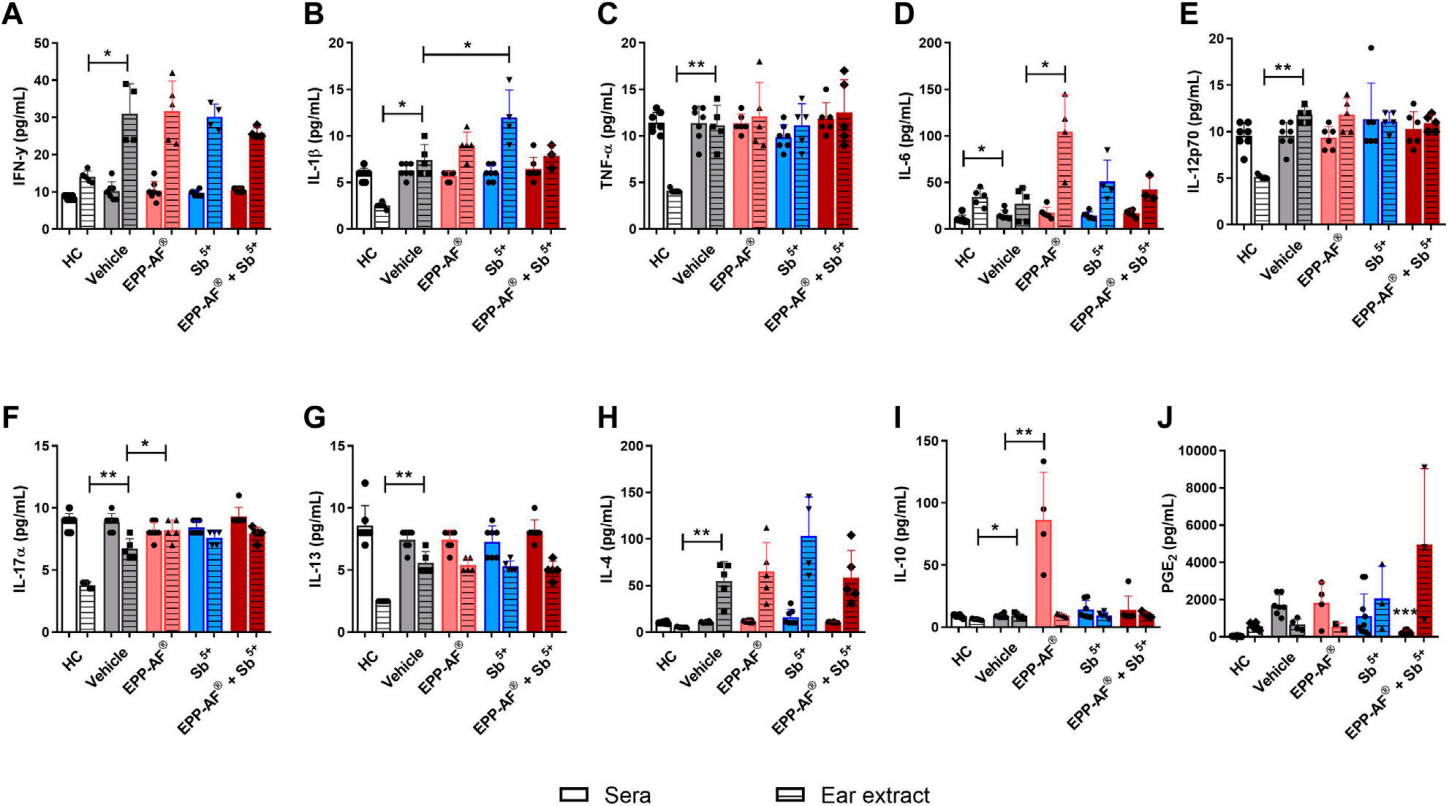
Inflammatory mediator quantification both systemically and in infected mouse ears following EPP-AF^®^ gel treatment. BALB/c mice were infected in the dermis of the ear with *L. amazonensis* and treated daily with EPP-AF^®^ gel alone (EPP-AF^®^), with meglumine antimoniate (Sb^5+^) alone, or with a combined regimen of EPP-AF^®^ gel + meglumine antimoniate (EPP-AF^®^ + Sb^5+^). Carbopol gel without EPP-AF^®^ was used as an untreated control (Vehicle). The ears and sera of uninfected animals were used as healthy controls (HC). At 7 weeks post-infection, animals were euthanized by cardiac puncture to collect sera; the animals’ ears were collected and macerated in liquid nitrogen to obtaining cellular extract, as described in Materials and Methods. Inflammatory mediator levels were determined by multiplex assay: **(A)** IFN-y, **(B)** IL-1β, **(C)** TNF-α, **(D)** IL-6, **(E)** IL-12p70, **(F)** IL-17α, **(G)** IL-13, **(H)** IL-4, **(I)** IL-10, **(J)** PGE_2_. Bars represent ±SD of one representative experiment performed with five animals per group (mediator levels quantification in mouse ears) or seven animals per group (mediator levels quantified in sera). The Kruskal-Wallis test, followed by Dunn’s post-test were used for multiple comparisons and the Mann-Whitney test was applied for comparison between two groups (*p < 0.05, **p < 0.01).

We observed that *L. amazonensis* infection in BALB/c mice led to the enhanced local production (i.e., at the site of infection and treatment) of several mediators involved in the inflammatory process and control of *Leishmania* infection (Figure 11). We found that treatment with EPP-AF^®^ gel alone significantly increased the production of IL-6 and IL-17α (Figures 11D, F, respectively), while the intraperitoneal application of meglumine antimoniate (Sb^5+^) resulted in enhanced IL-1β production (Figure 11B). Interestingly, no significant differences were observed in the ears of mice treated with combined (EPP-AF^®^ + Sb^5+^) therapy compared to untreated controls. In addition, our analysis of inflammatory mediators in the sera of untreated *L. amazonensis*-infected animals revealed increased levels of IL-6 in comparison to healthy controls (Figure 11D). Moreover, treatment with EPP-AF^®^ gel induced higher levels of IL-10 compared to untreated infected mice (Figure 11I), while combined treatment (EPP-AF^®^ + Sb^5+^) significantly reduced PGE_2_ levels (Figure 11J) in relation to untreated infected animals, partly corroborating our *in vitro* findings (Figure 6D).

## Discussion

The chemical composition of propolis can be affected by several factors, such as climate, season, flora visited by bees, geographic area and extraction method, which in turn directly influence the observed pharmacological properties (Menezes, 2005). To overcome these constrains, here we employed two formulations of Brazilian green propolis derived from standardized propolis extract (EPP-AF^®^), produced and characterized by Apis Flora Ltda., in order to ensure reproducibility across batches (Berretta et al., 2012). The present study investigated the effects of Brazilian green propolis EPP-AF^®^ against *L. amazonensis* infection *in vitro*, as well as in a chronic and highly inflammatory experimental murine model of Cutaneous Leishmaniasis (CL) (De Oliveira and Barral-NETO, 2005; Silva-Almeida et al., 2010).

Under *in vitro* analysis, an IC_50_ value of 23 ± 4 μg/mL was found for EPP-AF^®^, suggesting moderate activity (Indrayanto et al., 2021). Although Glucantime^®^ was not shown to be effective in reducing *L. amazonensis* intracellular viability at concentrations ranging from 1,000–10 μg/mL, which already provides evidence of low activity, the data in the literature indicate variable IC_50_ values for this compound, with results varying between 222.31 μg/mL - 22.2 μg/mL for intracellular amastigotes of different *Leishmania* species (Morais-Teixeira et al., 2008; Mirzaei et al., 2019; Mostafavi et al., 2019). The present IC_50_ result for EPP-AF^®^ serves to support findings in the literature regarding the leishmanicidal effects of propolis.

The control of CL is dependent on the induction of a Th1-type immune response involving the production of pro-inflammatory cytokines, notably IL-12, IFN-γ, IL-1α and TNF-α, which leads macrophage activation. Activated macrophages induce the expression of iNOS enzyme which, in turn, catalyzes L-arginine into nitric oxide (NO), a molecule that exerts intracellular microbicidal activity. In addition, CD8^+^ T-cells contribute to CL control through cytotoxic activity and the production of cytokines. On the other hand, susceptibility to CL has been linked to a regulatory Th2 type immune response, characterized by the production of high levels of regulatory and/or anti-inflammatory cytokines (such as IL-10, IL-4, and IL-13). Th2 cytokines induce the activation of the arginase I pathway and consequent biosynthesis of polyamines, thereby contributing to parasite proliferation and persistence (Abdoli et al., 2017). While a Th1 response contributes to the control of *Leishmania* infection, an exacerbated Th1 response can lead to severe tissue damage, such as that seen in CL (Banerjee et al., 2016; Abdoli et al., 2017; Gonçalves-de-Albuquerque et al., 2017; Gonzalez et al., 2020).

We observed an effective and balanced innate immune response following *in vitro* treatment with Brazilian green propolis (EPP-AF^®^), as evidenced by high levels of the TNF-α inflammatory cytokine and HO-1 antioxidant enzyme, in addition to reduced NO, PGE_2_ and IL-1β production in diverse inflammatory contexts. However, treatment with EPP-AF^®^ did not modulate the expression of arginase or ODC, which are considered potential therapeutic targets for CL due to the relationship of these enzymes with infection exacerbation. Discordantly, a previous study involving an experimental model of uveitis observed higher arginase levels in retinal biopsies of animals treated with propolis (Touri et al., 2018). Although iNOS expression was not modulated in infected cells, treatment with Brazilian green propolis EPP-AF^®^ did seem to inhibit the activity of this enzyme by reducing nitrite levels in LPS-activated cells, as well as INF-γ-activated macrophages regardless of infection. Importantly, some studies have demonstrated the ability of different propolis extracts, or their main components, to reduce NO levels in several inflammatory models, e.g., in the presence of LPS, by cells irradiated with ultraviolet B rays, and in cells from celiac patients (Touri et al., 2018; Alanazi et al., 2020; Yoshino et al., 2021).

Brazilian green propolis (EPP-AF^®^) treatment was found to increase the expression (and also appears to enhance the activity) of the antioxidant enzyme HO-1 in BMDM regardless of infection with *L. amazonensis*. Some studies have found HO-1 induction to be associated with parasite survival and increased susceptibility to *Leishmania* infection, as evidenced by the inhibition of important microbicidal mediators, such as nitric oxide (NO), reactive oxygen species (ROS) and TNF-α (Luz et al., 2012; Malta-Santos et al., 2017; Silva et al., 2020). However, the higher expression of this enzyme seen herein suggests the antioxidant potential of Brazilian green propolis EPP-AF^®^, which may contribute to inflammation control in CL similarly to what has been demonstrated in *Trypanosoma cruzi* (Gutierrez et al., 2014). In addition, studies investigating extracts or natural compounds that exert leishmanicidal activity, such as quercetin, which is present in propolis, have demonstrated the induction of nuclear factor erythroid-2-related factor2 (Nrf2) expression, as well as greater HO-1 expression. Higher levels of Nrf2 and HO-1 lead to increased ferritin expression, which then binds to labile iron, thus reducing availability for intracellular parasites and consequently promoting parasite death (Tomiotto-Pellissier et al., 2018; Cataneo et al., 2019; Miranda-Sapla et al., 2019).

Despite increases in COX-2 expression following EPP-AF^®^ treatment, our data suggest that this inflammatory enzyme’s activity becomes attenuated due to its ability to reduce levels of PGE_2_ (in response to COX-2 activation), since the effect of NS398, an inhibitor of COX-2 activity, was potentiated in *L. amazonensis*-infected BMDM. Concordantly, *in silico* analysis demonstrated the ability of a Uruguayan propolis extract, as well as its main phenolic compounds, to inhibit COX-1 and COX-2 enzymes (Paulino et al., 2016), and a reduction in PGE_2_ levels was observed in the supernatant of propolis-treated macrophages exposed to LPS or arachidonic acid (Rossi et al., 2002). The reduction in PGE_2_ levels observed herein is promising, given this lipid mediator’s association with parasite survival (Lonardoni et al., 1994; Venturin et al., 2020). To our knowledge, this work represents the first attempt to evaluate the *in vitro* effect of propolis extract on PGE_2_ production in the context of *Leishmania* infection.

Furthermore, significantly higher levels of TNF-α cytokine were observed following EPP-AF^®^ treatment in association with the activation of the ERK 1/2 cell signaling pathway. The ability of propolis to modulate TNF-α may represent a potential strategy for intracellular parasite killing, since this cytokine plays an important role in the control of *Leishmania* infection and is known to participate in clinical outcome (Rodrigues et al., 2021; Taraghian et al., 2021). Non-etheless, the role of TNF-α in the leishmanicidal effect exerted by EPP-AF^®^ deserves further investigation. Furthermore, the phosphorylation and activation of Nrf2 by ERK 1/2 can also lead to the expression of anti-oxidative genes, such as HO-1 enzyme (de Menezes et al., 2019). The induction of COX-2 expression has also been associated with the ERK 1/2 signaling pathway in human endometriosis stromal cells, as well as in colorectal cell cancer (Elder et al., 2002; Huang et al., 2013). Thus, it is plausible that the ERK 1/2 pathway may be involved in the higher expression of COX-2 and HO-1 seen in cells treated with Brazilian green propolis.

Considering that free propolis extract is commonly used in experimental models of leishmaniasis, the development of pharmaceuticals offering greater stability and preparations permitting controlled release, or even increased compound bioavailability, constitutes a substantial therapeutic advantage. Here, we successfully employed a gel formulation of Brazilian green propolis that allowed for gradual and prolonged propolis release for pre-clinical testing in a model of Cutaneous Leishmaniasis caused by *L. amazonensis*. Brazilian green propolis EPP-AF^®^ carbomer gel has already been successfully used in a preclinical model of vulvovaginal infection, showing antifungal action similar to clotrimazole cream (Berretta et al., 2013).

In BALB/c mice, *L. amazonensis* infection is characterized by the development of progressive, chronic, highly inflammatory parasitic lesions (De Oliveira and Barral-Neto, 2005). This mouse strain’s susceptibility to infection by *L. amazonensis* has made it a popular choice for efficacy studies evaluating new candidates for Cutaneous Leishmaniasis treatment. This murine model of infection allowed us to investigate the effectiveness of topical treatment with Brazilian green propolis gel alone, as well as in combination with meglumine antimoniate, in the reduction of lesion size in BALB/c mice dermally infected with *L. amazonensis* in the ear. We observed significant reductions in lesion size in animals treated with either EPP-AF^®^ alone or in combination with antimonial therapy. However, we found no differences in parasite load or inflammatory status in the treated mice, even under combinatorial treatment with Glucantime^®^. One reason for this finding could be the late time of immunologic profiling in our analyses (10 weeks after infection). Other studies have demonstrated the ability of different propolis extracts or their main chemical constituents to induce wound healing. Romana-Souza et al., 2018 reported the effectiveness of caffeic acid in promoting pressure ulcer healing in an experimental model, observing differences in the modulation of iNOS, NO, TNF-α, and NFκB at earlier times of analysis compared to later timepoints. A combined treatment of propolis and a nitric oxide donor ruthenium complex was also shown to be effective in reducing lesion size in mice infected with *L. amazonensis*; the modulation of NO, IL-10, and TGF-β levels, as well as cell recruitment, were all observed after 30 days of treatment (Miranda et al., 2015). It is important to note that, although none of the three treatment conditions evaluated herein reduced parasite load, this fact does not detract from the potential of EPP-AF^®^ as a promising candidate for adjuvant therapy, since the criteria considered for achieving cutaneous leishmaniasis cure are based on clinical findings, as defined by the complete epithelialization of all lesions and the disappearance of crusting, desquamation, infiltration and erythema, in contrast to sterile cure (i.e., complete parasite eradication) (da Saúde et al., 2017).

While several reports have demonstrated the leishmanial efficacy of Glucantime^®^ *in vivo*, herein we did not observe reductions in parasite load in our *in vivo* murine model of *L. amazonensis*. However, our data does corroborate previous reports detailing the ineffectiveness of Glucantime^®^ in reducing parasite load in *L. amazonensis-*infected BALB/c mice inoculated in the ear or footpad (Cos et al., 2018; Cavalcanti de Queiroz et al., 2019; Valentim Silva et al., 2020). Furthermore, it is important to highlight the divergent results in the scientific literature regarding important issues, such as dosage, quantity of inoculated *Leishmania* (10^5^–10^7^), and the model of cutaneous leishmaniasis employed (ear pinna, hind footpad or tail base), all of which may influence disease severity and outcome, as well as pharmacologic response (Loeuillet et al., 2016; Paladi et al., 2017; Coelho et al., 2016; Kauffmann et al., 2018). Most studies that have observed reduced parasite burden employed daily doses between 16.8 mg/Sb^5+^/kg/day and 200 mg/Sb^5^+/kg/day, or once a week at 500 mg/Sb^5+^/kg/week (Paladi et al., 2017; Brustolin et al., 2022; Muñoz-Durango et al., 2022). Herein, Glucantime^®^ was administered at a dose of 50 mg/Sb^5+^/kg/day to treat animals infected with 10^6^ stationary promastigotes in the ear dermis, which was designed to ensure the development of highly parasitized, non-healing and inflamed lesions. Although this drug did demonstrate statistical significance compared to the vehicle control (*p* < 0.05), it is plausible that, in this type of ear dermis infection model, a dose higher than 50 mg/Sb^5+^/kg/day could improve the observed effectiveness in reducing lesion size, and perhaps even parasite load, at the site of infection.

Considering that the prolonged duration of leishmaniasis treatment is a key factor in increasing drug resistance reported in underdeveloped countries (Freitas-Junior et al., 2012; Rajasekaran and Chen, 2015), a combined treatment strategy involving the topical application of Brazilian green propolis EPP-AF^®^ gel shows promise in reducing treatment time and increasing therapeutic efficacy compared to meglumine antimoniate alone.

## Conclusion

The present results indicate the effectiveness of Brazilian green propolis EPP-AF^®^ extract and its gel formulation in the control of *in vitro L. amazonensis* infection, as well as in disease progression as assessed *in vivo.* The observed reductions in the production of most of the inflammatory mediators evaluated following EPP-AF^®^ treatment, together with increased expression of the antioxidant enzyme HO-1 and enhanced levels of TNF-α, which aids in controlling parasite load, suggests the potential of propolis extract in orchestrating an effective and balanced innate immune response in the treatment of Cutaneous Leishmaniasis (CL), a condition associated with an intense inflammatory process. Accordingly, we submit that Brazilian green propolis (EPP-AF^®^) warrants consideration in the development of potential new adjuvant pharmaceutical strategies designed to treat CL.

## Data Availability

The original contributions presented in the study are included in the article/supplementary material, further inquiries can be directed to the corresponding authors.
